# Pulmonary Involvement in Sjögren’s Syndrome: Correlations with Biomarkers of Activity and High-Resolution Computer Tomography Findings

**DOI:** 10.3390/jcm13041100

**Published:** 2024-02-15

**Authors:** Deiana Roman, Stela Iurciuc, Alexandru Caraba

**Affiliations:** 1 Second Department of Internal Medicine, “Victor Babeș” University of Medicine and Pharmacy, 300041 Timisoara, Romania; roman.deiana@umft.ro (D.R.); caraba.alexandru@umft.ro (A.C.); 2 Department of Cardiology, “Victor Babeș” University of Medicine and Pharmacy, 300041 Timisoara, Romania

**Keywords:** Sjögren’s syndrome, primary SS, tumor necrosis factor-α, ESSDAI score, focus score molecular mechanisms

## Abstract

(1) Background: Sjögren’s syndrome (SS) represents a systemic autoimmune disease whose pathophysiology has yet to be elucidated, though it is known that the inflammatory process encountered in SS is of a systemic nature, with cytokines representing the main mediators for tissue damage. (2) Aim of the study: The aim of the present study is to further the understanding of the link between interleukin serum levels, cytokine serum levels, HRCT findings and the Warrick score (as tools for the evaluation of pulmonary involvement) in patients with pSS. (3) Methods: The present study is a retrospective, observational one aimed at ascertaining the link between SS activity and its clinical implications, as well as how interleukin and TNF-α levels correlate with systemic changes. The study enrolled 112 patients with pSS and 56 healthy subjects, matched for age and gender, as a control group. pSS activity was assessed using the ESSDAI. Cytokine levels and leukocyte and lymphocyte counts were measured in both groups. The focus score was calculated for each patient, HRCT was performed to assess lung function, and the Warrick score was calculated. (4) Conclusions: HRCT revealed NSIP in 13 patients (59.09%) and UIP in 9 patients. The strongest positive correlation was identified upon analyzing the relation between IL-8 and the Warrick score (r = 0.9156, *p* < 0.00001), followed by a positive correlation between the score and IL-6 levels (r = 0.5738, *p* < 0.0052). Unsurprisingly, the degree and severity of pulmonary involvement was also positively correlated with the degree of disease activity (r = 0.4345, *p* = 0.0433).

## 1. Introduction

Sjögren’s syndrome (SS) represents a systemic autoimmune disease whose pathophysiology has yet to be elucidated, though there are numerous factors that have been incriminated in the apparition of this pathology, namely autoimmune processes (that have been initiated and maintained over a longer period of time), environmental factors and triggers, as well as genetic predisposition [1]. It has been widely acknowledged that autoimmune diseases are more frequently encountered within the female population [2], and primary Sjögren’s syndrome (pSS) is no exception: perimenopausal women represent the main group affected by SS, with studies showing a ratio of up to 14:1 for female to male patients [3].

Patients with SS experience a plethora of clinical manifestations, depending on the extent of the inflammatory process, though they are mainly represented by exocrine gland dysfunctions. Sicca symptoms appear as a consequence of the inflammation of the salivary glands and lacrimal glands due to lymphocytic infiltration. This is clinically expressed as xerostomia and keratoconjunctivitis [4], with the chief clinical manifestations present in patients with SS.

There is a growing body of evidence showing that the inflammatory process encountered in SS is of a systemic nature, extending to other organs and systems, namely the lungs, the nervous system, lymph nodes, the skin and even the renal system [5]. The SS type present in a patient (either primary or secondary SS) can be established based on the coexistence of another autoimmune disease (secondary SS), or lack thereof (primary SS) [6]. Tissue damage occurs by mechanisms characteristic of the autoimmune responses that are promoted by cytokines. The cytokines involved in these processes derive from both immune cells such as T cells, B cells, NK cells, macrophages and monocytes, as well as non-immune cells such as fibroblasts, endothelial and epidermal cells [7].

An up-regulation of cytokines has been observed in target tissues and circulatory systems of patients with SS, including TNF-α and interleukins, which have a critical role to play in the pathogenesis of SS [8].

Tumor necrosis factor-α (TNF-α) plays a pivotal role in the pathogenesis of numerous autoimmune diseases, having a significantly higher production than in individuals who do not present with such a disease. The pathogenesis of SS is promoted by the role TNF-α has in stimulating the production of proinflammatory cytokines, in the recruitment of inflammatory cells and immune cells to the targeted tissues, as well as in the direct destruction of the tissues themselves. It has been observed that in patients living with SS, the elevated expression of TNF-α can be found in both their serum and salivary glands, unlike the TNF-α levels encountered in patients that had sicca syndrome without having SS [9,10].

Being an autoimmune disease that is complex in its nature and presents with systemic consequences, the European SS Disease Activity Index (ESSDAI) scoring system was conceived in order to better ascertain the degree of disease activity and provide healthcare personnel with a measurable means of evaluating patients with SS [11]. Furthermore, in order to diagnose the oral component of SS, a focus score (counting the number of mononuclear cells infiltrates on a 4 mm^2^ glandular section) of over 1 is necessary, with this score being the most important one in establishing the diagnosis [12]. As pulmonary involvement is one of the most common types of extra glandular manifestations, it is necessary for the clinician to evaluate pulmonary function on a regular basis, herein lying the value of an indicator of the extent and severity of the pulmonary affliction, such as the Warrick score [13].

The aim of the present study is to further the understanding of the link between interleukin serum levels, cytokine serum levels, HRCT findings and the Warrick score (as tools for the evaluation of pulmonary involvement) in patients with pSS.

## 2. Materials and Methods

### 2.1. Study Design

The present study is an observational, retrospective one that was performed in the Department of Internal Medicine, Division of Rheumatology, Timisoara, Romania, between September 2018 and July 2023. The study was conducted in accordance with the Declaration of Helsinki, and the study protocol was approved by the Ethical Committee of the Railway Hospital in Timisoara.

### 2.2. Patient Enrollment

The participants were represented by 112 consecutive patients with pSS and 56 healthy subjects, matched for age and gender, which comprised the control group. All the patients with pSS fulfilled the 2016 ACR/EULAR Classification Criteria for pSS [14].

Exclusion criteria were as follows: age under 18 years, lack of capacity to give informed consent, secondary Sjogren syndrome, overlap syndromes, sicca symptoms related to other diseases (sarcoidosis, IgG4-related disease, hepatitis C infection, acquired immunodeficiency syndrome, previous head and neck radiation therapy, graft versus host disease, uncontrolled diabetes mellitus, amyloidosis), acute inflammatory pulmonary diseases in 30 days before the investigation, chronic pulmonary diseases, pregnancy or breastfeeding women, chronic kidney disease (eRFG < 60 mL/min/1.73 m^2^), current tobacco consumers and use and/or abuse of drugs that might decrease salivary gland function.

The control group comprised healthy individuals admitted to the Internal Medicine Department for regular medical visits. Before enrolling in this study, all the patients with pSS and the patients in the control group gave their written informed consent.

Medical history and current treatment regimens (only in the case of patients with pSS) were documented for all the participants in this study. Complete clinical examination was performed for all the SS patients and the control group.

### 2.3. Statistical Analysis

We created a database that included anthropometric data, patient diagnosis, as well as hematologic and biochemical determinations, alongside results from high-resolution computed tomography, as well as patient scores regarding disease diagnosis, activity and lung implication.

The collected data were analyzed using the SPSS statistical software package, version 27 (IBM Corp, Armonk, NY, USA).

The Kolmogorov–Smirnov test was utilized for assessing the normal distribution of obtained data. Data found to have a normal distribution are presented as mean ± standard deviation. Statistical analyses were performed using parametric tests, such as ANOVA and Pearson’s correlation, in order to ascertain whether the investigated variables were linked, and if so, to what degree and outcome. The results were presented as correlations coefficients and significance levels. The threshold of statistical significance for this study was considered to be achieved if the value of *p* was under 0.05.

pSS activity was evaluated using EULAR Sjögren’s Syndrome Disease Activity Index (ESSDAI), encompassing twelve domains: constitutional, lymphadenopathy, glandular, articular, cutaneous, pulmonary, renal, muscular, peripheral nervous system, central nervous system, hematologic and biologic. An ESSDAI value below 5 represents a low level of disease activity, a value between 5 and 13 represents a moderate level of disease activity, while a value of over 14 is indicative of a high level of disease activity [15].

Antinuclear antibodies (ANAs), anti-SSA antibodies, anti-SSB antibodies, anti-RNP 70 antibodies, rheumatoid factor, serum beta-2 microglobulin, TNF-α, IL-6, IL-8, leukocytes and lymphocyte serum levels were evaluated in all patients presenting with pSS. Serum beta-2 microglobulin, TNF-α, IL-6, IL-8, leukocytes and lymphocytes were also evaluated in the control group.

Antinuclear antibodies were detected using indirect immunofluorescence (HELMED), while anti-SSA, anti-SSB and anti-RNP 70 antibodies were determined using a fluoroimmunoenzymatic assay. The rheumatoid factor was detected by latex agglutination test. Serum beta-2 microglobulin levels were assessed through the usage of immuno-enzymometric assay with chemiluminescence detection (CLIA-serum, limit of detection 24.7 pg/mL). Cytokine levels (TNF-α, IL-8 and IL-6) were evaluated using the chemiluminescence method (CLIA-serum, limit of detection 1.7 pg/mL, 90 fg/mL), as well as the electrochemiluminescence method (ECLIA-serum, limit of detection 0.04 pg/mL) by determining anti-cytokine autoantibodies (ACAAs).

The focus score assessment: The focus score represents one of the elements used in the American College of Rheumatology/European League Against Rheumatism classification criteria for primary Sjögren’s syndrome. This score is calculated based on the results of the histopathological exams of the minor salivary glands. These glands are readily accessible for biopsy, with their placement under the inner surface of the lip facilitating this procedure. After the administration of 10% Lidocaine so as to provide local anesthesia, between 5 and 7 minor glands were biopsied from the inner surface of the inferior lip. Hematoxylin-eosin staining was used in order to assess the tight clumps of lymphocytes (≥50), the so-called foci. Their density on a surface of 4 mm^2^ was used to calculate the focus score. A focus score above 1 was considered to be positive [16].

High-resolution computed tomography: In order to detect the degree and severity of these patients’ pulmonary involvement, a high-resolution computed tomography (HRCT) scan of the chest was performed (Siemens CT Definition Edge 128; Siemens, München, Germany).

The HRCT scans were performed at peak inspiration visualizing the lungs from their apex to their base in a supine position, with slice thickness of 1.5 mm. Upon performing the HSCRT, the following represented the focus of attention: ground-glass opacities, consolidations, septal/subpleural lines (including parenchymal bands and reticular pattern), irregular pleura, nodules, subpleural cysts, architectural distortions, traction bronchiectasis, honeycombing, atelectasis, pleural effusion and mosaic attenuation patterns [17].

Pulmonary involvement was quantified according to the Warrick score. The score was calculated by the summation of the scores for five basic radiological interstitial lung disease findings (ranging from 0 to 5) and the extent of these changes (ranging from 0 to 3). The values of this score vary between 0 and 30, with a minimum score of 7 having been needed in order to predict a pulmonary interstitial disease [18].

## 3. Results

The present study included a group of patients with SS comprising 112 patients, of which there were 74 female patients (66.07%) and 38 male patients (33.92%), as well as a control group comprising 56 patients, of which there were 36 female patients (64.28%) and 20 male patients (35.71%). The mean age of patients within the SS group was of 55.73 ± 7.08 years, similar to that of the control group (54.19 ± 5.89 years).

The demographic characteristics of the patients with pSS, as well as those of the patients in the control group, can be found in Table 1.

Patients in the pSS group underwent different treatment regimens, either monotherapy or variations of molecule associations, with the following drugs used: 78.57% underwent treatment with Hydroxychloroquine, 38.39% were treated with Azathioprine, 40.17% were treated with Mycophenolate mofetil, 10.71% underwent treatment with Cyclophosphamide, and 60.71% had Corticoids included in their treatment regimens. A summarization of treatment options prescribed to the study group is included in Table 2.

Both ANA and rheumatoid factor were identified in all the pSS patients included in the study group, while they were not found in any of the participants enrolled in the control group. Among antinuclear antibodies, anti-SSA and anti-SSB were present (anti-SSA abs. 85.41 ± 56.43 u/mL, anti-SSB abs. 65.65 ± 54.88 u/mL), but anti-centromere and anti-RNP 70 antibodies were not identified in the studied patients.

SS activity was assessed by using the ESSDAI score system (the mean value was 12.08 ± 7.53). Based on the ESSDAI values, the patients with pSS were classified as having low disease activity (if the ESSDAI score was <5), moderate disease activity (if the ESSDAI score was between 5 and 13) and high disease activity (if the ESSDAI was ≥14). In total, 22 patients were found to have low disease activity, while the rest (90 patients) were equally distributed between the moderate disease activity and high disease activity groups (45 patients in each category). The focus score of these pSS patients was 4.03 ± 1.60, indicating that the overwhelming majority of patients with pSS also presented with the oral component of the disease.

Both TNF-α and IL-6 were increased in patients with pSS, compared to patients enrolled in the control group (27.82 ± 17.78 vs. 7.80 ± 4.74, *p* < 0.0001, and 32.68 ± 15.14 vs. 6.79 ± 5.12, *p* < 0.0001, respectively). Serum beta-2-microglobulin, a marker of lymphocyte activity, was also increased in the pSS study group versus the control group (3.39 ± 1.02 vs. 1.92 ± 0.46, *p* < 0.01).

The values of both leukocytes and lymphocytes were reduced in the patients with pSS, when compared to the healthy control group (3085.75 ± 1101.01 vs. 7585.94 ± 1227.08, *p* < 0.001, and 749.56 ± 368.51 vs. 2224.01 ± 419.34, *p* < 0.001, respectively). A summary of the aforementioned data is presented in Table 3.

Raynaud’s phenomenon was identified in 25 patients (22.32%) of the pSS group of patients, as well as in 4 patients from the control group (7.14%), achieving statistical significance (*p* < 0.01), proving that while it can occur in healthy individuals, Raynaud’s phenomenon is typically encountered in those with other autoimmune pathologies.

Pulmonary disease of varying degrees was identified in 22 patients of the pSS group (19.64%) and was absent from the control group. The main clinical manifestations of the pulmonary disease at the time of diagnosis were represented by dyspnea (17 cases, 77.27%), dry cough (20 cases, 90.90%), chest tightness (10 cases, 45.45%) and recurrent pulmonary infections (four cases, 18.18%).

It was observed that patients with pSS and pulmonary disease were more advanced in age (*p* < 0.0001) and had longer history of disease duration (*p* < 0.05).

It was also found that patients with higher pulmonary involvement rates presented higher disease activity scores as evaluated by the ESSDAI compared to patients with lower rates of pulmonary involvement, with a statistically significant difference (*p* < 0.0001).

From a biological standpoint, patients with pSS and pulmonary involvement presented higher TNF-α levels (*p* < 0.001), higher IL-6 (*p* < 0.001) and IL-8 levels (*p* < 0.001), as well as higher serum leukocyte (*p* < 0.001) and lymphocyte levels (*p* < 0.01), values that were significantly higher than in their pSS counterparts who did not present pulmonary involvement.

The differences between patients with diagnosed pSS associated with pulmonary affliction and those without are summarized in Table 4.

High-resolution pulmonary computed tomography (CT) revealed non-specific interstitial pneumonia (NSIP) in 13 patients (59.09%) and usual interstitial pneumonia (UIP) pattern in 9 patients (40.90%). Warrick scores were higher in UIP pattern than in NSIP pattern (27.66 ± 1.11 versus 15.07 ± 2.84, *p* < 0.00001).

A strong positive correlation can be seen between pulmonary interstitial and IL-8 serum levels (r = 0.9156, *p* < 0.00001, Pearson’s correlation test). A positive correlation, even though not as strong, could be observed between pulmonary interstitial disease and IL-6 serum levels (r = 0.5738, *p* < 0.0052, Pearson’s correlation test). Another statistically significant correlation can be observed in relation to beta-a-microglobulin (r = 0.4357, *p* = 0.0426, Pearson’s correlation test). This leads to the assumption that patients exhibiting higher levels of IL-8, IL-6 and beta-2-microglobulin will present with higher rates of pulmonary involvement.

The extent and severity of interstitial pulmonary disease was also significantly and positively correlated with disease activity as measured by the ESSDAI score (r = 0.4345, *p* = 0.0433, Pearson’s correlation test) and the focus score (r = 0.4379, *p* = 0.0415, Pearson’s correlation); these findings indicate that pulmonary involvement is more common and extensive in those with oral pSS, as well as in those with higher disease activity.

Regarding leukocyte and lymphocyte serum levels, the present study’s findings indicate that a significant, moderate and negative correlation exists between the Warrick score and them (r = −0.4280, *p* = 0.0469, and r = −0.5525, *p* = 0.0076, respectively), signifying that in those with more extensive pulmonary involvement, both leukocytes and lymphocytes tend to decrease.

Surprisingly, the Warrick score’s correlation with pSS patient’s age and disease duration did not reach the threshold for statistical significance (r = 0. 1823, *p* = 0.4167, and r = 0.0716, *p* = 0.7515, respectively), and neither did the correlation with TNF-α (r = 0.3382, *p* = 0.1236).

A summary of the correlations between the Warrick score describing the extent and severity of pulmonary involvement and the monitored parameters can be found in Table 5.

**Figure 1:**
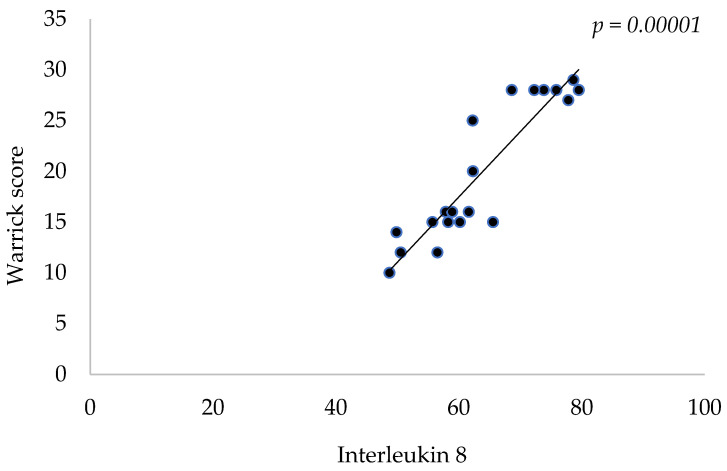
Correlation between Warrick score and IL-8.

Among the studied parameters, the extent and severity of pulmonary involvement was most strongly correlated with IL-8 values (r = 0.9156, *p* < 0.00001), indicating that high serum IL-8 was encountered in patients with the highest pulmonary disease severity and extent (Figure 1).

**Figure 2:**
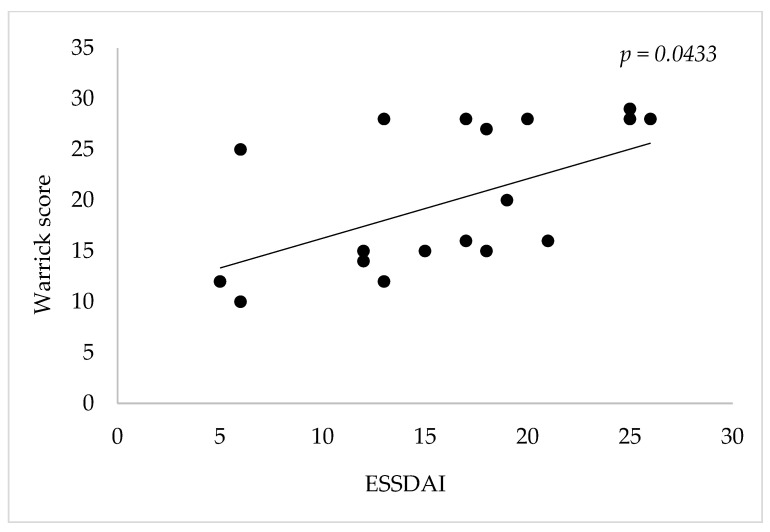
Correlation between Warrick score and ESSDAI.

Disease activity evaluated by means of the ESSDAI score was found to have a low positive correlation with the severity and extent of pulmonary involvement as evaluated by the Warrick score (r = 0.4345, *p* = 0.0433), suggesting that the higher the disease activity, the more compromised the pulmonary function is (Figure 2).

## 4. Discussion

The present study is a retrospective, observational one aimed at ascertaining the link between SS activity and its clinical implications, as well as the modality in which interleukin and TNF-α levels correlated with systemic changes.

To date, the connection between cytokines and the phenotype of SS has been successfully studied, though the connection between cytokine levels and disease activity remains somewhat unclear. Taking this into consideration, it comes as no surprise that no specific treatment is available to patients with SS, and studies that centered around the implementation of cytokine-targeted therapies have not yielded positive outcomes [19,20].

We have found IL-6 to be significantly more elevated in patients with SS, results that are congruent with the existing literature [1]. Further research is warranted in determining how blocking this interleukin might benefit patients through blocking the development of the disease, with studies involving mouse models having already proven promising, even though human studies have not been able to replicate those results [21,22].

Among the patients enrolled in the SS group with pulmonary involvement, high-resolution pulmonary CT was performed, and it revealed that 59.09% presented with NSIP and 40.90% had UIP, with Warrick scores being higher in patients with a UIP pattern than in those with an NSIP pattern (27.66 ± 1.11 versus 15.07 ± 2.84, *p* < 0.00001). Those with pulmonary involvement were found to have had a longer duration of SS (*p* < 0.05), and to be more advanced in age (*p* < 0.0001), as well as higher disease activity scores (*p* < 0.0001) and cytokine values (*p* < 0.001). The main clinical manifestations of the pulmonary disease in the studied SS group of patients were dyspnea (17 cases, 77.27%), dry cough (20 cases, 90.90%), chest tightness (10 cases, 45.45%) and recurrent pulmonary infections (4 cases, 18.18%), similar to manifestations reported by G. Stojan et al. [23]. These findings are in accord with the existing studies, with one study encountering a percentage of 19.83% of SS patients with ILD [24] (compared to the 19.62% found in our study), while others stipulate a percentage between 9 and 20% [25].

In the present study, we have utilized the Warrick score for evaluating the severity and extent of pulmonary involvement in patients with SS, with results showing a positive correlation between the score and IL-6 levels (r = 0.5738, *p* < 0.0052). We have not been able to find literature confirming or contradicting this finding in patients with SS, although papers correlating IL-6 levels and alveolitis extent have been published regarding patients with systemic sclerosis [26,27], and as such, this makes a compelling case for IL-6 to be considered in the context of a patient with SS and a high Warrick score.

The strongest positive correlation was identified upon analyzing the relation between IL-8 and the Warrick score (r = 0.9156, *p* < 0.00001), signifying that high levels of IL-8 are indicative of a more severe and extensive pulmonary involvement. This correlation between cytokine levels and disease activity has also been reported by other scientific papers, though IL-8 was not the focus of the aforementioned publications [28,29], underlining the need for further research in regard to utilizing interleukins as a means for monitoring disease activity in relation to the lung, as well as a more in-depth delving into the etiological mechanisms of SS.

Unsurprisingly, the degree and severity of pulmonary involvement was also positively correlated with the degree of disease activity (r = 0.4345, *p* = 0.0433), with recently published papers ascertaining the frequency in which pulmonary involvement is present in those with SS, while urging healthcare professionals to actively search for and closely monitor the evolution of this systemic manifestation of the disease [30].

Among the strengths of this study, it is worth noting that it is the first of its kind to have been conducted in the Western part of Romania, as well as being the first in Romania. Given the country’s specifics regarding its population, namely health literacy, socio-economic development in the general aspect, alongside the extent of late presentations in the hospital and late diagnosis that ensues, we believe this study will be of great value for health practitioners in the field of family medicine, general medicine, pneumology and rheumatology. The results of the study could enable the early diagnosis of pulmonary involvement in these patients, leading to better disease management, and a better quality of life. Another strongpoint of the present study is the lack of overlap between different autoimmune diseases, guaranteeing that the results are specific to patients with SS, with the observed results not being due to any kind of confounding.

Regarding possible weaknesses of the present study, one could argue that a bigger sample size might be required, or even that the specific traits of the Romanian population limit the applicability of the findings, though we consider them applicable to at least the entirety of Eastern Europe. Another possible weakness could reside in the homogeneity of the studied group (exclusively Caucasian), limiting the knowledge and applicability of the findings to other ethnic groups.

In regard to further research in this field, we aim towards extending the patient database with the aid of our Romanian colleagues, to better ascertain the national burden of this pathology and its implications for the population, as well as its prevalence and incidence among other ethnic groups and minorities.

## 5. Conclusions

HRCT revealed NSIP in 13 patients (59.09%) and UIP in 9 patients. The strongest positive correlation was identified upon analyzing the relation between IL-8 and the Warrick score (r = 0.9156, *p* < 0.00001), followed by a positive correlation between the score and IL-6 levels (r = 0.5738, *p* < 0.0052). Unsurprisingly, the degree and severity of pulmonary involvement was also positively correlated with the degree of disease activity (r = 0.4345, *p* = 0.0433) and leukocytes.

## Figures and Tables

**Table 1:** Demographic characteristics of studied patients and controls.

Parameter	SS Patients	Control	*p*-Value
Number of patients:	112	56	
Female	74 (66.07%)	36 (64.28%)	>0.05
Male	38 (33.92%)	20 (35.71%)	
Age (years)	55.53 ± 7.08	54.19 ± 5.89	>0.05
Disease duration (years)	7.58 ± 3.94	-	-

**Table 2:** Drugs utilized in the treatment of patients with pSS.

Treatment	Hydroxy Chloroquine	Azathioprine	Mycophenolate Mofetil	Cyclophosphamide	Corticoids
Number of patients	88	43	45	12	68

**Table 3:** Biological findings in SS patients and control group.

Parameter	SS Patients	Control	*p*-Value
Focus score	4.03 ± 1.60	-	-
ESSDAI	12.08 ± 7.53	-	-
Anti-SSA abs	85.41 ± 56.43	-	-
Anti-SSB abs.	65.65 ± 54.88	-	-
TNF-α (pg/mL)	27.82± 17.78	7.80 ± 4.74	<0.0001
IL-6 (pg/mL)	32.68 ± 15.14	6.79 ± 5.12	<0.0001
IL-8 (pg/mL)	47.13 ± 16.20	13.89 ± 5.87	<0.0001
Beta-2-microglobulin (mg/L)	3.39 ± 1.02	1.92 ± 0.46	<0.01
Leukocytes/mm^2^	3083.75 ± 1101.01	7586.94 ± 1227.08	<0.001
Lymphocytes/mm^2^	749.56 ± 368.51	2224.01 ± 419.34	<0.001

**Table 4:** SS with and without pulmonary involvement.

Parameter	SS with Pulmonary Involvement	SS without Pulmonary Involvement	*p*-Value
Mean age (years)	61.54 ± 5.31	54.06 ± 6.69	<0.0001
Disease duration (years)	9.72 ± 4.24	7.06 ± 3.70	<0.05
Focus score	5.09 ± 1.65	3.77 ± 1.48	<0.01
ESSDAI	17.31 ± 7.22	10.81 ± 7.07	<0.0001
Anti-SSA abs.	120.78 ± 57.12	76.76 ± 53.07	<0.01
Anti-SSB abs.	72.96 ± 64.37	63.86 ± 52.55	>0.05
TNF-α	41.71 ± 19.78	24.42 ± 15.58	<0.001
IL-6	39.78 ± 15.91	24.98 ± 13.73	<0.001
IL-8	63.98 ± 9.66	42.17 ± 13.72	<0.001
Beta-2-microglobulin	4.11 ± 1.17	3.22 ± 0.91	<0.01
Leukocytes	2394.18 ± 909.57	3252.32 ± 1081.84	<0.001
Lymphocytes	513.68 ± 354.34	807.22 ± 350.18	<0.01

**Table 5:** Correlations between Warrick score and monitored parameters.

Parameter	r	*p*-Value
Mean age (years)	0.1823	0.4167
Disease duration (years)	0.0716	0.7515
Focus score	0.4379	0.0415
ESSDAI	0.4345	0.0433
TNF-α	0.3382	0.1236
IL-6	0.5738	0.0052
IL-8	0.9156	0.00001
Beta-2-microglobulin	0.4357	0.0426
Leukocytes	−0.4280	0.0469
Lymphocytes	−0.5525	0.0076
